# Supplementary material for: Populism and health. An evaluation of the effects of right-wing populism on the COVID-19 pandemic in Brazil
Source: PLoS One. 2022 Dec 13;17(12):e0269349. doi: 10.1371/journal.pone.0269349 (PMC9747047; doi:10.1371/journal.pone.0269349)
Supplement: S1 Appendix — (DOCX) [file pone.0269349.s001.docx]

**Populism and health. An evaluation of the effects of right-wing populism on the COVID-19 pandemic in Brazil**

We detail data sources, descriptive statistics, and results in this appendix.

**Data Sources and Descriptive Statistics**

We drew on an original dataset covering Brazil’s 5,570 municipalities to evaluate the connection between right-wing extremism and the spread of the pandemic using ecological data from electoral results. Table 01 shows Descriptive Statistics.

Brazil is a federal country comprised of 27 states and 5,570 municipalities. We use data on COVID-19 results collected by BRASIL-IO (2020). Detailed data on cases of infections and deaths because of COVID-19 is published daily by Brazilian state authorities. Brasil-IO is a non-government organization that collects data from the states and publishes data at local and national levels. It daily compiles epidemiological bulletins from the 27 State Health Departments and provides a database with the historical series of confirmed cases and deaths by municipalities. Using state-collected data is of fundamental importance since the federal government, on several occasions, has taken measures to jeopardize the transparency of the Brazilian Ministry of Health.

We collected votes for the most successful right-wing candidate in the 2014 presidential elections on the TSE (Electoral Superior Court), as well as data on Bolsonaro's share of the vote in 2018. The Electoral Superior Court is part of the Brazilian Judiciary Branch, which oversees and organizes elections. The institution has detailed data about all elections in Brazil.

We measure Social Isolation compliance using geo-located data from cell phones (Inloco, 2020). InLoco® is a Brazilian technology company that provides intelligence that collects anonymous location data from devices. It monitors more than 60 million mobile phones in the country in association with mobile companies. Individual information is not shared, only a municipality's average levels of social isolation. The *Social Isolation Index* was developed by In Loco® and is publicly accessible to help combat the Covid-19 pandemic in Brazil, characterizing the collection of secondary data. The startup's software measures the index through the percentage of mobile devices that remained within a radius of 450 meters from the location identified as home.

We collected data on cases of infections caused by severe acute respiratory syndrome (SARS) in 2018, Hospital Beds, and Additional Hospital Beds added in 2020 on the Health Ministry database (DATASUS).

Data on SARS, a viral respiratory disease caused by a SARS-associated coronavirus, has been collected weekly by the Brazilian government since 2009 when the H1N1 pandemic happened. The database covers all cases of severe acute respiratory syndrome observed in the country. Every healthcare unit in the country must report cases to the Minister of Health. After 2020, it also included COVID-19. We use all cases in 2018 of viral respiratory disease caused by a SARS-associated coronavirus. 2018 was the last year available.

We collected the size of the local economy, population, the share of the population older than 60 years, child mortality, life expectancy, the distance to Brasilia, the federal capital, and the distance to the relevant state capital from the Brazilian Bureau of Statistics and Geography, IBGE.

The Brazilian Ministry of Education calculates the Education Index of Vulnerability. It synthesizes the characteristics of individuals concerning their income, occupation, and education based on annual data collected from all students in every municipality. It is a proxy for the general vulnerability of families.

**Detailed Results**

Table 2 shows **panel data estimates** for weekly deaths and confirmed cases of infection. Bolsonaro's local share of votes is statistically significant in all models. The larger the share of votes he received, the more cases and deaths were seen. The incidence rate ratio indicates that an increase of 1 percentage point in Bolsonaro's vote share would show a corresponding increase in the municipal rate for cases by a factor of 1.002 in the first model to 1.005 in model 4, in which we include all controls. Although relevant, these numbers are smaller than the figures found by Hawkins, Charles, and Mehaffey (2020) for socio-economic status. They found that the impact of age older than 65 increases the risk of having the disease by 1.070, being black increases by 1,030, and not having a high school degree increases by 1.100.

It is interesting to note that the traditional right-wing vote has a negative impact on cases of infections, decreasing the municipal rate of cases by a factor of 0.994. That implies that traditional conservative areas were less affected than more recently right-wing populist areas in both the number of cases of infection and deaths.

Regarding basic controls, social isolation has a mixed effect. Although it may seem counter-intuitive, isolation may have happened as a reaction to cases and deaths and not just the other way around. There may be self-selection in the isolation compliance process, as the municipalities that adopt stricter isolation policies and whose residents are more sensitive to the call for social isolation are the most affected by the disease.

Hospital beds are statistically significant in reducing the rates of deaths. Additional hospital beds nevertheless showed no impact, suggesting that health supply improvements were ineffective in dealing with the disease. Both of them, as expected, does not affect cases. The size of the local economy seems to be an essential indicator of cases, not deaths, underlying how economic factors may also determine between those who would recover and those who would die from the disease. Distance from the state capital is irrelevant to the number of life losses but seems to predict fewer cases.

On the other hand, places more distant from the federal capital Brasilia have no consistent effect. However, they are associated with more deaths when we use all controls, which could mean that health care is less effective. Eventually, as expected, larger municipalities and older populations have more cases and deaths.

Table 3 shows **cross-sectional findings concerning** all respiratory disease cases during 2018 and the total number of cases and deaths from COVID-19 in 2020. Findings show that Bolsonaro's vote share is not statistically significant when we use control variables. Besides, the traditional right-wing vote is not associated with SARS, which strongly indicates that politics does affect overall respiratory diseases before the pandemic; the cases of SARS were not affected by distance from the state or federal capital either. Larger and richer municipalities were also more affected than the others. However, regarding COVID-19 outcomes, Bolsonaro’s share of the vote in 2018 has a statistically significant impact, as shown in Table 2, associated with more cases and deaths.

In Table 04A and Table 04B, we present the results when considering a population weighting measure. We use rates of cases and deaths for 1000 inhabitants. The results are the same.

Finally, Table 5 explores **the effect of support for Bolsonaro on social distancing** as a likely mechanism. Once again, results indicate that the populist right-wing vote is associated with lower compliance with social isolation, whereas the traditional right-wing vote does not. We estimate that a ten percentage-point increase in Bolsonaro's vote share decreases social isolation on average by 2.6 percentage points.

Lastly, columns 7 and 8 in Table 5 present the traditional mediation analysis proposed by Baron and Kenny (1986), where the effects of Bolsonaro's vote share on the log of deaths per capita can be partially explained by the effect of poor social distancing. The partial effects of Bolsonaro's vote share are smaller and less significant whenever we introduce the social isolation index into the model, which is strongly significant and consistent with the mediation analysis adopted. Imai et al. (2011) approach confirms the results.

Once more, the traditional right-wing vote is not correlated with compliance with social isolation and is negatively correlated with deaths, indicating that the disease affected fewer areas with the traditional right-wing vote.

*Bayesian mediation analysis*

Mediation analysis is a reasonable approach for examining causal mechanisms that underlie a relationship between a postulated independent and a dependent variable by including a third mediation variable. Rather than suppose a direct causal relationship, a causal mediation model shows that an independent variable influences a dependent one indirectly through a mediator. We estimate the indirect effect by the average causal mediation effect (ACME). The average direct effect (ADE) estimates the direct effect and represents all possible mechanisms, including a non-mediated relationship. The total effect (TE) is the sum of the ACME and the ADE. We employ a two-stage mediation method estimated by Bayesian multilevel models, as Imai et al. (2011) proposed.

We use multilevel linear regressions to estimate the effect of Bolsonaro’s share of the vote on the inverted social isolation compliance, measured by the '*1 – social isolation index*’ of Inloco (2020), and to estimate the effects of the votes for Bolsonaro on the log of the number of deaths per capita. In both models, the intercept varies by municipality. All models contain all controls listed in Table 2 of the manuscript (Log of the size of the local economy and Log Population; Hospital Beds, Additional Hospital Beds in 2020, Share Pop. Older than 60 years, Distance to Federal and State Capital, Child Mortality Rate, Life Expectancy in 2000, and Educational Vulnerability) and state and week fixed effects.

We opt to invert the social isolation index, as both relations should become positive. Bolsonaro’s share of the vote should have a positive impact on the inverted social isolation index and also on the per capita number of deaths, and the inverted social isolation index should have a positive impact on the per capita number of deaths.

By the Bayesian mediation analysis approach of Imai et al. (2011), we estimate that 21.79% of the effect of the vote for Bolsonaro in 2018 on deaths is mediated by the decline in compliance with social isolation. The results are in Table 4 of the manuscript.

**Table 1 Descriptive Statistics**

| Variable | Obs. | Mean | Std. Dev. | Min | Max |  |
| --- | --- | --- | --- | --- | --- | --- |
| Weekly Covid Cases | 5,570 | 63.545 | 345.852 | 0 | 16607 |  |
| Weekly Deaths due to Covid | 5,570 | 1.213 | 9.299 | 0 | 480 |  |
| Confirmed cases in the year | 5,570 | 1367.03 | 7916.473 | 2 | 401718 |  |
| All Deaths due to Covid in the year | 5,570 | 34.912 | 324.955 | 0 | 15679 |  |
| Cases of Respiratory Diseases in 2018 | 5,570 | 8.701 | 97.102 | 0 | 4227 |  |
| Bolsonaro’s Share of the Vote in 2018 | 5,570 | 38.726 | 18.982 | 1.941 | 83.893 |  |
| Right Wing Share of the Vote in 2014 | 5,570 | 32.806 | 17.34 | 1.514 | 82.562 |  |
| Annual Social Isolation Compliance Average | 4,778 | 39.636 | 3.322 | 24 | 56.604 |  |
| **Controls**  Hospital Beds | 5,570 | 53.872 | 320.064 | 0 | 14822 |  |
| Additional Hospital Beds | 5,570 | 5.459 | 51.275 | 0 | 2586 |  |
| Log Local Wealth | 5,570 | 12.309 | 1.405 | 9.472 | 20.366 |  |
| Log Population | 5,570 | 2.568 | 1.172 | -0.208 | 9.402 |  |
| Share Pop. Older than 60 years | 5,565 | 0.176 | .05 | 0.034 | .435 |  |
| Log Distance to Federal Capital | 5,507 | 6.871 | .525 | 0 | 7.961 |  |
| Log Distance to State Capital | 5,507 | 5.258 | .878 | 0 | 7.297 |  |
| Child Mortality Rate per 1.000 | 5,570 | 12.889 | 12.821 | 0 | 181.820 |  |
| Life Expectancy | 5,570 | 66.982 | 0.509 | 54.35 | 78,10 |  |
| Education Index of Vulnerability | 5,547 | 4.726 | 0.509 | 3.514 | 5.952 |  |
|  | | | | | | |

| **Table 2. Analysis of the relationship between ideology, isolation, and other responses from COVID-19 – panel data** | | | | | | | | | | |
| --- | --- | --- | --- | --- | --- | --- | --- | --- | --- | --- |
|  | (1) | (2) | (3) | (4) |  | (5) | (6) | (7) | (8) |  |
|  | Weekly Cases | | | |  | Weekly Deaths | | | |  |
| Bolsonaro’s Share of the Votes in 2018 | 0.002 | 0.012 | 0.005 | 0.005 |  | 0.007 | 0.023 | 0.012 | 0.008 |  |
|  | [0.000]*** | [0.000]*** | [0.001]*** | [0.001]*** |  | [0.001]*** | [0.001]*** | [0.001]*** | [0.001]*** |  |
| Right Wing Share of the Vote in 2014 |  | -0.013 | -0.003 | -0.005 |  |  | -0.022 | -0.007 | -0.010 |  |
|  |  | [0.000]*** | [0.001]*** | [0.001]*** |  |  | [0.001]*** | [0.001]*** | [0.001]*** |  |
| Weekly Social Isolation Average with one lag |  |  |  | -0.025 |  |  |  |  | -0.031 |  |
|  |  |  |  | [0.001]*** |  |  |  |  | [0.002]*** |  |
| Weekly Social Isolation Average with two lags |  |  |  | 0.032 |  |  |  |  | 0.038 |  |
|  |  |  |  | [0.001]*** |  |  |  |  | [0.002]*** |  |
| Hospital Beds |  |  | 0.000 | 0.000 |  |  |  | 0.000 | 0.000 |  |
|  |  |  | [0.000] | [0.000] |  |  |  | [0.000]*** | [0.000]*** |  |
| Additional Hospital Beds |  |  | 0.000 | 0.000 |  |  |  | 0.000 | 0.000 |  |
|  |  |  | [0.000] | [0.000] |  |  |  | [0.000] | [0.000] |  |
| Log Local Wealth |  |  | 0.038 | 0.046 |  |  |  | -0.037 | -0.002 |  |
|  |  |  | [0.008]*** | [0.009]*** |  |  |  | [0.020] | [0.021] |  |
| Log Population |  |  | 0.093 | 0.086 |  |  |  | 0.417 | 0.363 |  |
|  |  |  | [0.009]*** | [0.011]*** |  |  |  | [0.023]*** | [0.025]*** |  |
| Share Pop. Older than 60 years |  |  |  | 1.186 |  |  |  | 0.205 | 1.537 |  |
|  |  |  |  | [0.119]*** |  |  |  | [0.253] | [0.252]*** |  |
| Log Distance to Federal Capital |  |  | 0.056 | -0.022 |  |  |  | -0.039 | 0.047 |  |
|  |  |  | [0.017]*** | [0.011] |  |  |  | [0.020]* | [0.016]** |  |
| Log Distance to State Capital |  |  | -0.032 | -0.078 |  |  |  | 0.010 | -0.006 |  |
|  |  |  | [0.004]*** | [0.004]*** |  |  |  | [0.008] | [0.008] |  |
| Child Mortality Rate per 1.000 |  |  | -0.001 | 0.000 |  |  |  | 0.000 | -0.001 |  |
|  |  |  | [0.000]** | [0.000] |  |  |  | [0.001] | [0.001] |  |
| Life Expectancy |  |  | 0.005 | 0.006 |  |  |  | -0.009 | -0.011 |  |
|  |  |  | [0.001]*** | [0.001]*** |  |  |  | [0.003]** | [0.003]*** |  |
| Education Index of Vulnerability |  |  | 0.180 | 0.205 |  |  |  | 0.303 | 0.163 |  |
|  |  |  | [0.024]*** | [0.025]*** |  |  |  | [0.058]*** | [0.048]*** |  |
| Dummies for States |  |  | x |  |  |  |  |  |  |  |
| Dummies for Brazilian Region |  |  |  | x |  |  |  | x |  |  |
| Constant | -1.091 | -1.077 | -3.007 | -3.062 |  | -0.796 | -0.841 | -2.713 | -2.821 |  |
|  | [0.008]*** | [0.008]*** | [0.192]*** | [0.148]*** |  | [0.026]*** | [0.026]*** | [0.306]*** | [0.268]*** |  |
| ln_r | -0.249 | -0.202 | -0.040 | -0.125 |  | 0.665 | 0.705 | 0.976 | 0.896 |  |
| _cons | [0.017]*** | [0.017]*** | [0.019]* | [0.021]*** |  | [0.022]*** | [0.022]*** | [0.025]*** | [0.027]*** |  |
| ln_s | 2.098 | 2.197 | 2.531 | 2.444 |  | -0.306 | -0.196 | 0.437 | 0.408 |  |
| _cons | [0.024]*** | [0.024]*** | [0.026]*** | [0.031]*** |  | [0.020]*** | [0.021]*** | [0.032]*** | [0.035]*** |  |
| N | 245,080 | 245,080 | 241,384 | 164,967 |  | 245,080 | 245,080 | 241,384 | 164,967 |  |
| Linear regression coefficients with standard errors in parentheses. *** p < 0.01, ** p < 0.05, and * p < 0.10, respectively. | | | | | | | | | | |
| SEs are clustered by states. | | | | | | | | | | |

| **Table 3. Analysis of the relationship between ideology, isolation, and other responses from COVID-19 – cross-sectional analysis** | | | | | | | | |
| --- | --- | --- | --- | --- | --- | --- | --- | --- |
|  | (1) | (2) | (3) | (4) |  | (5) |  | (6) |
|  | Severe Acute Respiratory Syndrome (SARS)  2018 Cases | | | |  | Covid - 2020 full Cases |  | Covid - 2020 full Deaths |
|  |  |  |  |  |  |  |  |  |
| Bolsonaro’s Share of the Votes in 2018 | 0.082 | 0.141 | 0.007 | 0.008 |  | 0.019 |  | 0.024 |
|  | [0.014]*** | [0.019]*** | [0.015] | [0.019] |  | [0.005]*** |  | [0.005]*** |
| Right Wing Share of the Vote in 2014 |  | -0.071 | 0.026 | 0.003 |  | -0.011 |  | -0.013 |
|  |  | [0.020]*** | [0.013]* | [0.011] |  | [0.005]* |  | [0.004]*** |
| Hospital Beds |  |  | 0.799 | 0.522 |  | 0.180 |  | 0.185 |
|  |  |  | [0.189]*** | [0.108]*** |  | [0.075]* |  | [0.040]*** |
| Additional Hospital Beds |  |  | 0.851 | 1.263 |  | 0.806 |  | 0.896 |
|  |  |  | [0.211]*** | [0.139]*** |  | [0.070]*** |  | [0.050]*** |
| Log Local Wealth |  |  |  | 0.000 |  | 0.000 |  | 0.000 |
|  |  |  |  | [0.000] |  | [0.000] |  | [0.000] |
| Log Population |  |  |  | -0.001 |  | -0.002 |  | 0.000 |
|  |  |  |  | [0.002] |  | [0.001] |  | [0.001] |
| Share Pop. Older than 60 years |  |  |  | 4.672 |  | -1.488 |  | 1.352 |
|  |  |  |  | [2.523] |  | [0.782] |  | [0.665]* |
| Log Distance to Federal Capital |  |  |  | -0.104 |  | 0.254 |  | 0.083 |
|  |  |  |  | [0.250] |  | [0.117]* |  | [0.107] |
| Log Distance to State Capital |  |  |  | 0.184 |  | -0.013 |  | -0.094 |
|  |  |  |  | [0.121] |  | [0.037] |  | [0.032]** |
| Child Mortality Rate per 1.000 |  |  |  | 0.013 |  | 0.002 |  | 0.002 |
|  |  |  |  | [0.009] |  | [0.001]** |  | [0.001] |
| Life Expectancy |  |  |  | 0.039 |  | -0.006 |  | 0.008 |
|  |  |  |  | [0.046] |  | [0.013] |  | [0.011] |
| Education Index of Vulnerability |  |  |  | 0.814 |  | -0.145 |  | -0.546 |
|  |  |  |  | [0.548] |  | [0.140] |  | [0.130]*** |
| Constant | -1.669 | -1.748 | -14.294 | -19.090 |  | 0.957 |  | -1.405 |
|  | [0.687]* | [0.729]* | [1.896]*** | [4.117]*** |  | [1.135] |  | [0.940] |
| lnalpha_cons | 3.093 | 3.045 | 1.604 | 1.526 |  | -0.975 |  | -1.390 |
|  | [0.117]*** | [0.098]*** | [0.172]*** | [0.166]*** |  | [0.070]*** |  | [0.082]*** |
| N | 5570 | 5570 | 5570 | 5486 |  | 5486 |  | 5486 |
| Linear regression coefficients with standard errors in parentheses. *** p < 0.01, ** p < 0.05, and * p < 0.10, respectively. | | | | |  |  |  |  |
| SEs are clustered by states. | | | | | | | | |

**Table 4A - Analysis of the relationship between ideology, isolation, and other responses from COVID-19 with offsetting variable**

|  | (1) | (2) |
| --- | --- | --- |
|  | Covid Confirmed cases per 1.000 inhabitant | Death Covid cases per 1.000 inhabitants |
| Bolsonaro’s Share of the Votes in 2018 | 0.012 | 0.000 |
|  | [0.004]*** | [0.000]*** |
| Right Wing Share of the Vote in 2014 | -0.002 | 0.000 |
|  | [0.003] | [0.000]*** |
| Weekly Social Isolation Average with one lag | -0.008 | 0.000 |
|  | [0.005] | [0.000] |
| Weekly Social Isolation Average with two lags | 0.016 | 0.000 |
|  | [0.006]* | [0.000]*** |
| Hospital Beds | 0.000 | 0.000 |
|  | [0.000] | [0.000] |
| Additional Hospital Beds | 0.000 | 0.000 |
|  | [0.001] | [0.000] |
| Log Local Wealth | 0.133 | 0.002 |
|  | [0.050]** | [0.001]*** |
| Log Population | -0.098 | -0.001 |
|  | [0.057] | [0.001] |
| Share Pop. Older than 60 years | -0.914 | 0.026 |
|  | [0.571] | [0.007]*** |
| Log Distance to Federal Capital | 0.059 | 0.001 |
|  | [0.062] | [0.001] |
| Log Distance to State Capital | -0.024 | -0.002 |
|  | [0.032] | [0.000]*** |
| Child Mortality Rate per 1.000 | 0.001 | 0.000 |
|  | [0.001] | [0.000] |
| Life Expectancy | -0.001 | 0.000 |
|  | [0.008] | [0.000] |
| Education Index of Vulnerability | -0.042 | -0.005 |
|  | [0.128] | [0.002]* |
| Dummies for Brazilian Region | X | X |
|  | [0.164]*** | [0.002] |
| Constant | -1.436 | -0.021 |
|  | [0.803] | [0.016] |
| N | 164,967 | 164,967 |
| Wald chi2(18) | 729.95 | 3543.04 |
| Prob > chi2 | 0.000 | 0.000 |
| Linear regression coefficients with standard errors in parentheses. *** p < 0.01, ** p < 0.05, and * p < 0.10, respectively. | | |
| SEs are clustered by states. |  |  |

**Table AB. Analysis of the relationship between ideology, isolation, and other responses from COVID-19 – cross-sectional analysis with offsetting variable.**

|  | (1) | (2) | (3) |
| --- | --- | --- | --- |
|  | SARS cases per 1000 inhabitants | Covid Confirmed cases per 1000 inhabitants | Death Covid cases per 1000 inhabitants |
| Bolsonaro’s Share of the Votes in 2018 | 0.000 | 0.547 | 0.014 |
|  | [0.001] | [0.136]*** | [0.002]*** |
| Right Wing Share of the Vote in 2014 | 0.000 | -0.284 | -0.008 |
|  | [0.001] | [0.143] | [0.002]*** |
| Hospital Beds | 0.000 | 0.008 | 0.000 |
|  | [0.000] | [0.007] | [0.000] |
| Additional Hospital Beds | 0.001 | -0.042 | 0.000 |
|  | [0.000] | [0.042] | [0.001] |
| Log Local Wealth | 0.027 | 4.971 | 0.095 |
|  | [0.007]*** | [2.433] | [0.020]*** |
| Log Population | 0.026 | -5.631 | -0.050 |
|  | [0.010]* | [2.270]* | [0.023]* |
| Share Pop. Older than 60 years | 0.228 | -66.795 | 0.744 |
|  | [0.195] | [31.248]* | [0.387] |
| Log Distance to Federal Capital | 0.001 | 8.204 | 0.023 |
|  | [0.014] | [3.801]* | [0.059] |
| Log Distance to State Capital | 0.016 | -0.282 | -0.065 |
|  | [0.009] | [1.390] | [0.021]** |
| Child Mortality Rate per 1.000 | 0.000 | 0.079 | 0.001 |
|  | [0.000] | [0.027]** | [0.001] |
| Life Expectancy | 0.000 | -0.081 | 0.002 |
|  | [0.001] | [0.341] | [0.006] |
| Education Index of Vulnerability | 0.044 | -3.939 | -0.271 |
|  | [0.024] | [5.021] | [0.062]*** |
| Constant | -0.726 | -47.396 | 0.425 |
|  | [0.270]* | [35.766] | [0.483] |
| N | 5,483 | 5,483 | 5,483 |
| F(12,26) | 31.600 | 8.030 | 93.270 |
| Prob > chi2 | 0.000 | 0.000 | 0.000 |
| Linear regression coefficients with standard errors in parentheses. *** p < 0.01, ** p < 0.05, and * p < 0.10, respectively. | | | |
| SEs are clustered by states. |  |  |  |

| **Table 5. Analysis of the relationship between right-wing populism and social isolation** | | | | | | | | | | |
| --- | --- | --- | --- | --- | --- | --- | --- | --- | --- | --- |
|  | Weekly Social Isolation Average | | |  | Average Isolation in the Year | | |  | Log (Deaths per Weak) | |
|  | (1) | (2) | (3) |  | (4) | (5) | (6) |  | (7) | (8) |
| Bolsonaro’s Share of the Votes in 2018 | -0.069 |  | -0.026 |  | -0.070 |  | -0.026 |  | 0.004 | 0.003 |
|  | [0.007]*** |  | [0.010]* |  | [0.008]*** |  | [0.011]* |  | [0.001]*** | [0.001]** |
| Right Wing Share of the Vote in 2014 |  | -0.052 | -0.014 |  |  | -0.051 | -0.013 |  | -0.006 | -0.007 |
|  |  | [0.007]*** | [0.011] |  |  | [0.007]*** | [0.012] |  | [0.001]*** | [0.001]*** |
| Weekly Social Isolation Average with one lag |  |  |  |  |  |  |  |  |  | -0.031 |
|  |  |  |  |  |  |  |  |  |  | [0.002]*** |
| Hospital Beds |  |  | -0.001 |  |  |  | -0.001 |  | 0.000 | 0.000 |
|  |  |  | [0.000]** |  |  |  | [0.000]** |  | [0.000]*** | [0.000]*** |
| Additional Hospital Beds |  |  | 0.008 |  |  |  | 0.008 |  | -0.001 | -0.001 |
|  |  |  | [0.003]** |  |  |  | [0.003]** |  | [0.000]* | [0.001] |
| Log Local Wealth |  |  | -0.089 |  |  |  | -0.061 |  | 0.055 | 0.052 |
|  |  |  | [0.141] |  |  |  | [0.146] |  | [0.014]*** | [0.015]*** |
| Log Population |  |  | -0.380 |  |  |  | -0.460 |  | -0.405 | -0.410 |
|  |  |  | [0.222] |  |  |  | [0.235] |  | [0.016]*** | [0.017]*** |
| Share Pop. Older than 60 years |  |  | -0.047 |  |  |  | 0.088 |  | -0.006 | -0.017 |
|  |  |  | [2.142] |  |  |  | [2.194] |  | [0.187] | [0.205] |
| Log Distance to Federal Capital |  |  | 2.357 |  |  |  | 2.474 |  | -0.013 | 0.057 |
|  |  |  | [0.890]** |  |  |  | [0.917]* |  | [0.029] | [0.031] |
| Log Distance to State Capital |  |  | -0.358 |  |  |  | -0.366 |  | -0.051 | -0.062 |
|  |  |  | [0.127]** |  |  |  | [0.128]** |  | [0.008]*** | [0.009]*** |
| Child Mortality Rate per 1.000 |  |  | 0.002 |  |  |  | 0.003 |  | 0.000 | 0.000 |
|  |  |  | [0.006] |  |  |  | [0.007] |  | [0.001] | [0.001] |
| Life Expectancy |  |  | -0.034 |  |  |  | -0.036 |  | 0.000 | 0.000 |
|  |  |  | [0.025] |  |  |  | [0.028] |  | [0.002] | [0.003] |
| Education Index of Vulnerability |  |  | -1.516 |  |  |  | -1.505 |  | -0.062 | -0.108 |
|  |  |  | [0.540]** |  |  |  | [0.569]* |  | [0.042] | [0.047]* |
| State Dummies | X | X | X |  | X | X | X |  | X | X |
| Epidemiological Week Dummies |  |  |  |  |  |  |  |  | X | X |
| Constant | 46.435 | 44.287 | 39.012 |  | 46.480 | 44.270 | 38.113 |  | 0.898 | 2.181 |
|  | [0.390]*** | [0.246]*** | [8.765]*** |  | [0.431]*** | [0.256]*** | [9.119]*** |  | [0.326]** | [0.343]*** |
| N | 173742 | 173742 | 172846 |  | 4778 | 4778 | 4747 |  | 171027 | 171027 |
| Linear regression coefficients with standard errors in parentheses. *** p < 0.01, ** p < 0.05, and * p < 0.10, respectively. | | | | | | | | | | |
| SEs are clustered by states. | | | | | | | | | | |

**REFERENCES**

Baron, Reuben; David Kenny. 1986. The Moderator - Mediator Variable Distinction in Social Psychological Research." Journal of Personality and Social Psychology 51(6):1173.

Brasil.IOBrasil.IO: COVID-19 epidemiological bulletins by municipality per day, available at: https://brasil.io/dataset/covid19/. Source: Health Departments of the Federative Units, data treated by Álvaro Justen and others. 2020

Hawkins, Robert B., Eric J. Charles, and J. Hunter Mehaffey. "Socio-economic status and COVID-19–related cases and fatalities." Public health 189 (2020): 129-134.

Imai, Kosuke; Luke Keele, Dustin Tingley, Teppei Yamamoto (2011) Unpacking the Black Box of Causality: Learning about causal mechanisms from experimental and observational studies. American Political Science Review 105(4):765–789.

InLoco. Brazilian COVID-19 Map.2020. Índice de Isolamento Social: Brasil. 2020 Available from:https://public.tableau.com/profile/inloco.tableau#!/vizhome/MKTScoredeisolamentosocial/VisoGeral.
